# Foregut separation and tracheo-oesophageal malformations: The role of tracheal outgrowth, dorso-ventral patterning and programmed cell death

**DOI:** 10.1016/j.ydbio.2009.11.005

**Published:** 2010-01-15

**Authors:** Adonis S. Ioannides, Valentina Massa, Elisabetta Ferraro, Francesco Cecconi, Lewis Spitz, Deborah J. Henderson, Andrew J. Copp

**Affiliations:** a Neural Development Unit, UCL Institute of Child Health, London, UK; b Dulbecco Telethon Institute - IRCCS Fondazione Santa Lucia and Department of Biology, University of Rome “Tor Vergata”, Rome, Italy; c Surgery Unit, UCL Institute of Child Health, London, UK; d Institute of Human Genetics, Newcastle University, Newcastle upon Tyne, UK

**Keywords:** Mouse, Embryo, Trachea, Oesophagus, Tracheo-oesophageal defects, Malformations, Apoptosis, Anoikis, Cell proliferation, Adriamycin

## Abstract

Foregut division—the separation of dorsal (oesophageal) from ventral (tracheal) foregut components—is a crucial event in gastro-respiratory development, and frequently disturbed in clinical birth defects. Here, we examined three outstanding questions of foregut morphogenesis. The origin of the trachea is suggested to result either from respiratory outgrowth or progressive septation of the foregut tube. We found normal foregut lengthening despite failure of tracheo-oesophageal separation in Adriamycin-treated embryos, whereas active septation was observed only in normal foregut morphogenesis, indicating a primary role for septation. Dorso-ventral patterning of Nkx2.1 (ventral) and Sox2 (dorsal) expression is proposed to be critical for tracheo-oesophageal separation. However, normal dorso-ventral patterning of Nkx2.1 and *Sox2* expression occurred in Adriamycin-treated embryos with defective foregut separation. In contrast, *Shh* expression shifts dynamically, ventral-to-dorsal, solely during normal morphogenesis, particularly implicating Shh in foregut morphogenesis. Dying cells localise to the fusing foregut epithelial ridges, with disturbance of this apoptotic pattern in Adriamycin, *Shh* and *Nkx2.1* models. Strikingly, however, genetic suppression of apoptosis in the *Apaf1* mutant did not prevent foregut separation, indicating that apoptosis is not required for tracheo-oesophageal morphogenesis. Epithelial remodelling during septation may cause loss of cell-cell or cell-matrix interactions, resulting in apoptosis (anoikis) as a secondary consequence.

## Introduction

Oesophageal atresia (OA) and tracheo-oesophageal fistula (TOF) are common foregut malformations, affecting around 1 in 3500 births and frequently requiring emergency surgery in the neonatal period (Shaw-Smith, 2006). While advances have been made in understanding the genetic aetiology of syndromic forms of OA/TOF (Garcia-Barcelo et al., 2008; Van Bokhoven et al., 2005; Vissers et al., 2004; Williamson et al., 2006), the majority of cases are non-syndromic and their cause is unknown (Ioannides and Copp, 2009). Moreover, the mechanisms underlying the embryonic and fetal development of OA/TOF are poorly understood.

The oesophagus and trachea develop from a single embryonic structure: the anterior foregut tube. The respiratory primordium appears during development when the laryngo-tracheal groove emerges from the ventral aspect of the post-pharyngeal foregut. Caudal elongation of the laryngo-tracheal groove along the undivided foregut generates the laryngeal and tracheal primordia. Bifurcation and subsequent branching of the posterior-most aspect of the respiratory primordium gives rise to the bronchi and lungs. Shortly after appearance of the broncho-pulmonary bifurcation, the dorsal, oesophageal part of the foregut tube begins to separate from the ventral, tracheal component, with a wave of morphogenesis travelling in a caudal-to-cranial direction along the foregut. Separation occurs in the human embryo between Carnegie stages 13 and 16 (28–37 days post-fertilisation), and between embryonic days (E) 11 and 12 in the mouse.

While the early events of tracheo-oesophageal separation are difficult to study in human embryos and fetuses, information has emerged from a teratogenic model of OA/TOF based on exposure of mid-gestation rat embryos to Adriamycin (doxorubicin). This anthracycline antibiotic enters the nucleus and intercalates into DNA, interfering with DNA replication and transcription (Diez-Pardo et al., 1996). We adapted the Adriamycin teratogenic model for use in the mouse, in order to facilitate molecular and genetic studies (Ioannides et al., 2002, 2003). Partial or complete failure of separation of the respiratory and gastrointestinal foregut components was observed in 47% of Adriamycin-treated mouse embryos and fetuses. In the present study we compared the embryonic pathogenesis of OA/TOF in Adriamycin-treated mice, and in mice lacking function of sonic hedgehog (Shh) or Nkx2.1. *Shh* null embryos exhibit severe tracheo-oesophageal malformations (Litingtung et al., 1998; Pepicelli et al., 1998), as do mice with mutations in genes downstream from Shh, namely *Gli2^−/−^; Gli3^+/−^* double mutants (Motoyama et al., 1998) and *Foxf1* heterozygotes (Mahlapuu et al., 2001). In addition, mice null for *Nkx2.1* also develop OA/TOF, with a phenotype closely resembling the human malformation (Minoo et al., 1999).

The present study addresses three distinct questions relating to the morphogenetic mechanisms that underlie foregut separation, and which are disrupted in OA/TOF. First, we studied the growth dynamics of the developing foregut in order to distinguish between two alternative models of early tracheal morphogenesis. According to the ‘septation’ model, foregut division occurs when paired epithelio-mesenchymal ridges, arising from the lateral aspects of the foregut tube, fuse to form a septum. This separates the foregut lumen into dorsal (gastrointestinal) and ventral (respiratory) components (Qi and Beasley, 2000). Although often considered the most likely mechanism for tracheo-oesophageal separation, direct evidence of septum formation has been lacking (Kluth et al., 1987; O'Rahilly and Muller, 1984; Sasaki et al., 2001; Zaw-Tun, 1982). An alternative ‘tracheal outgrowth’ hypothesis considers the trachea to develop as a result of rapid growth of the respiratory primordium away from the foregut tube (Sasaki et al., 2001; Zaw-Tun, 1982). This model does not require the existence of lateral ridges or a septum, and is in keeping with the development of other foregut derivatives. For example, the thyroid, thymus and parathyroid glands bud off the foregut and grow rapidly away from it, eventually losing their original foregut connections.

The second aim of the present study was to determine whether loss of dorso-ventral gene expression patterning in the foregut tube is a pre-requisite for failure of tracheo-oesophageal separation. *Nkx2.1* and *Sox2* are expressed specifically in ventral and dorsal foregut endoderm, respectively, with a common dorso-ventral boundary separating the expression domains (Minoo et al., 1999; Que et al., 2007). In mice lacking *Sox2* function, OA/TOF coincides with ectopic (dorsal) expression of *Nkx2.1* in the foregut. Conversely, *Sox2* is ectopically expressed in OA/TOF resulting from *Nkx2.1* loss of function (Minoo et al., 1999; Que et al., 2007). It has been suggested, therefore, that disturbance of foregut dorso-ventral patterning may be a primary mechanism leading to failure of tracheo-oesophageal separation. We examined this idea further by determining whether dorso-ventral patterning is disrupted in the Adriamycin and *Shh*-null mouse models of OA/TOF.

The third focus of this paper relates to a possible requirement for programmed cell death (PCD) in foregut division. Several studies have identified dying cells in the region of tracheo-oesophageal separation, and have noted reduced apoptosis in Adriamycin-treated rat embryos (Orford et al., 2001; Qi and Beasley, 1998, 2000; Williams et al., 2000; Zhou et al., 1999). It has not been determined, however, whether this apoptosis is necessary for successful foregut separation or, alternatively, may be a secondary phenomenon. Here, we used a genetic approach to suppress embryonic apoptosis in order to determine the functional role of PCD in foregut separation.

## Materials and methods

### Mouse strains and Adriamycin administration

Inbred CBA/Ca mice (Harlan, UK) were used as the ‘normal’ strain in this study. Adriamycin (Pharmacia & Upjohn UK) was administered to pregnant females at 4 mg/kg body weight on both E7.5 and E8.5 (Ioannides et al., 2002). Saline was administered as a control. Embryos homozygous for a *Shh* null mutation were generated by matings between heterozygotes, as described (Chiang et al., 1996). *Apaf1* and *Nkx2.1* wild-type and null embryos were bred and genotyped as described (Cecconi et al., 1998; Tekki-Kessaris et al., 2001).

### Histology, immunohistochemistry and *in situ* hybridisation

Embryos were fixed in 4% paraformaldehyde and paraffin sections (5–10 μm thickness) were processed for Methyl Green or Periodic Acid Schiff (PAS) staining, immunohistochemistry (Ioannides et al., 2002) or RNA *in situ* hybridisation using digoxygenin-labelled riboprobes for *Shh* and *Sox2* (Avilion et al., 2003) as described previously (Breitschopf et al., 1992). Primary antibodies were: anti-Nkx2.1 (1:100, NeoMarkers MS-699-P1), anti-activated Caspase 3 (1:1000, Cell Signaling 9661S) and anti-Phosphohistone H3 (1:200, Upstate 06-570). Secondary antibodies were: biotinylated rabbit anti-mouse (DAKO E0354) and biotinylated goat anti-rabbit (DAKO E0432).

### Respiratory foregut length analysis

The respiratory foregut was identified by immunohistochemistry for Nkx2.1, a transcription factor specific for the respiratory system (Minoo et al., 1999). Its rostral and caudal limits and the level of tracheo-oesophageal separation were identified on transverse sections. The length of individual foregut segments was calculated by counting the number of corresponding sections of known thickness.

### Cell proliferation and programmed cell death (PCD) analysis

Data for calculation of mitotic and PCD indices were collected from sections at different rostro-caudal levels and individual dorso-ventral foregut zones. At E10.5, levels 1 and 4 were defined respectively as the rostro-caudal positions of the laryngotracheal groove and emerging lung buds, while levels 2 and 3 were evenly spaced between these levels. At E11.5, levels 1, 3, 6 and 7 were defined as the rostro-caudal positions of the laryngotracheal groove, point of tracheo-oesophageal separation, tracheal bifurcation, and separated bronchi and oesophagus. Levels 2, 4, and 5 were evenly interpolated between these levels. The spatial pattern of PCD was further analysed using a templating technique (Sharma et al., 2004) in which apoptotic cells from sections of corresponding rostro-caudal levels from different embryos are superimposed onto a single template section. This method can reveal spatial patterns of cell death that may not be obvious from observation of small numbers of apoptotic cells in individual sections.

### Graphical and statistical analysis

Quantitative data were represented by vertical box plots prepared using Sigmaplot (version 4.01). Vertical boxes with error bars define the median, 10th, 25th, 75th and 90th percentile values and all outlying values are represented by dots. Statistical comparisons (Sigmastat, version 1.0) were performed using the unpaired *t*-test (where data were normal) or the Mann–Whitney test (if normality failed). A *p*-value smaller than 0.05 was considered significant.

## Results

**Fig. 1:**
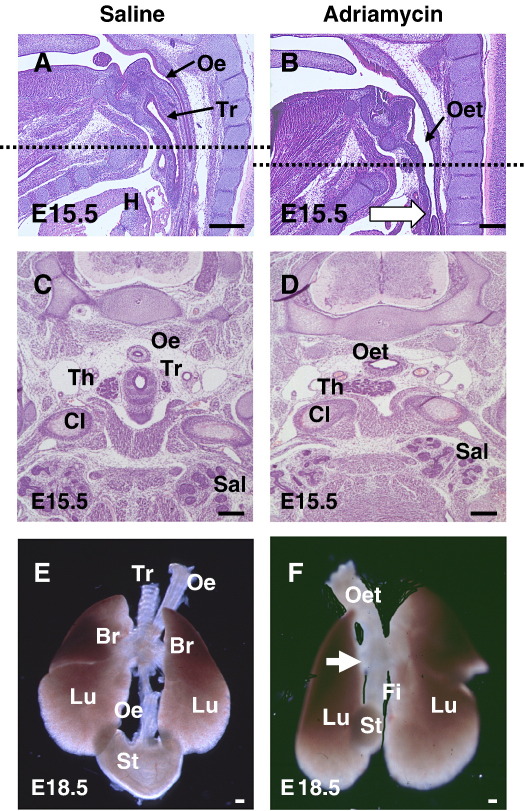
Structure of the foregut in normal and Adriamycin-treated mouse fetuses. (A–D) Sagittal (A, B) and transverse (C, D) sections stained with haematoxylin & eosin through E15.5 saline-treated (A, C) and Adriamycin-treated (B,D) fetuses. The level of the transverse sections in C and D corresponds to the level of the dashed lines in A and B (level of clavicular heads (Cl) and thoracic inlet). (E,F) Dissected anterior foregut structures from E18.5 saline-treated (E) and Adriamycin-treated (F) fetuses. After saline treatment (A, C, E), the oesophagus (Oe) and trachea (Tr) are separate structures in contrast to Adriamycin-treated fetuses (B, D, F) which have an undivided oesophagotrachea (Oet) at the same level. The fistula (Fi) arises from the oesophagotrachea at the level of the cardiac outflow tract (white arrow in B), forming a trifurcation with the main bronchi (white arrow in F), and connecting to a small, globular stomach (St). Note the prominent cartilaginous rings in the saline-treated trachea (E), whereas these appear completely absent from the Adriamycin-treated oesophagotrachea (F). Abbreviations: Br, bronchus; H, heart; Lu, lungs; Sal, salivary glands; Th, thyroid gland. Scale bars, 200 μm.

In the mouse, 40% of Adriamycin-treated embryos exhibit failure of foregut division, giving rise to a malformation closely resembling human OA/TOF. In normal E15.5 embryos, the trachea and oesophagus are present as distinct structures (Figs. 1A, C), whereas Adriamycin-treated embryos have an undivided oesophagotrachea (Figs. 1B, D). At E18.5, whole mount preparations demonstrate not only the lack of tracheo-oesophageal separation, but also the trifurcation at the level of the lung buds in Adriamycin-treated embryos, with a fistula connecting the oesophagotrachea to the globular stomach (Fig. 1F). In normal embryos, the oesophageal connection to the stomach is entirely separate from the tracheal connection to the lungs (Fig. 1E). There is a striking lack of cartilage development in the oesophagotrachea (Fig. 1F) compared with the normally separated trachea (Fig. 1E).

### Foregut growth during normal and abnormal tracheo-oesophageal separation

To investigate the mode of origin of the trachea, as a structure distinct from the oesophagus, we measured the length of the respiratory component of the foregut, and of its divided and undivided segments, between E10.5 and E12.5. The respiratory foregut, between the subglottic larynx and the tracheal bifurcation, was identified by immunohistochemistry for Nkx2.1 (also called TTF-1 and T/EBP), a transcription factor specific for the respiratory system (Minoo et al., 1999).

**Fig. 2:**
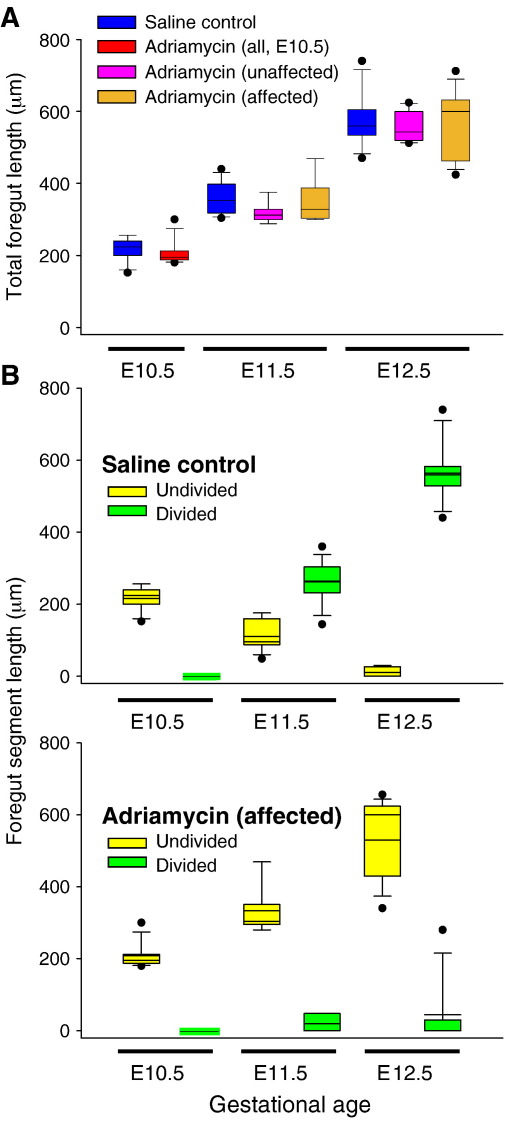
Respiratory foregut length analysis. Box plots showing total respiratory foregut length (A) and corresponding lengths of divided and undivided segments (B) according to gestational age. (A) Unaffected (normal phenotype) and affected (abnormal phenotype) Adriamycin-treated embryos are indistinguishable morphologically at E10.5, and their lengths are grouped together (red box). Note the closely similar overall foregut growth trajectories in all groups. (B) Lengths of undivided (yellow boxes) and divided (green boxes) foregut are shown for saline-treated controls (upper graph) and affected Adriamycin-treated embryos (lower graph). Unaffected Adriamycin-treated embryos have been omitted for clarity. Note the progressive division of the foregut in controls, but persistence of undivided foregut in affected Adriamycin-treated embryos.

The foregut is entirely undivided in normal E10.5 CBA/Ca embryos, whereas tracheo-oesophageal separation is underway at E11.5, and the trachea and oesophagus are largely separate structures by E12.5. While this morphogenetic activity is occurring, the respiratory foregut increases in length linearly (Fig. 2A, blue boxes). Strikingly, although the total length of the respiratory foregut increases significantly from E10.5 to E11.5 (Fig. 2A; *p* < 0.0001), the length of the undivided segment is significantly shorter at E11.5 than at E10.5 (yellow boxes in Fig. 2B, upper graph; *p* < 0.0001). At E12.5, the separation process is almost complete, as evidenced by the very short length of foregut that remains undivided (Fig. 2B upper graph). Hence, the further significant increase in total length of the respiratory foregut between E11.5 and E12.5 (Fig. 2A; *p* < 0.0001) is contributed to almost exclusively by growth of the separated trachea.

Following Adriamycin treatment, all embryos have an undivided foregut at E10.5, as in untreated CBA/Ca controls (Fig. 2A, red box). At E11.5 and E12.5, approximately 60% of Adriamycin-treated embryos also resemble controls, showing a partially (E11.5) or completely (E12.5) divided foregut. These unaffected embryos show a linear increase in total foregut length between E10.5 and E12.5 (Fig. 2A, pink boxes), with a lengthening of the divided segment and a shortening of the undivided segment in a manner closely similar to untreated controls (data not shown). In contrast, 40% of Adriamycin-treated embryos (*n* = 13/45 at E11.5; 12/20 at E12.5) have either a totally undivided foregut or only a very short divided segment. In these affected embryos, the divided segment remains significantly shorter than in control embryos at both E11.5 and E12.5, whereas the undivided segment is significantly longer (Fig. 2B lower graph; *p* < 0.0001). Nevertheless, the total foregut length increases linearly to the same extent in these embryos as in unaffected Adriamycin-treated and untreated control embryos (Fig. 2A, brown boxes).

In conclusion, while total respiratory foregut length increases linearly from E10.5 to E12.5, this is independent of tracheo-oesophageal separation: similar growth dynamics are observed in both affected Adriamycin-treated embryos and untreated controls. Moreover, the absolute length of undivided foregut decreases as the length of divided foregut increases in normal embryos. These findings strongly suggest a process of tracheo-oesophageal separation, progressively along the length of the foregut, but not a model based on specific outgrowth and lengthening of the respiratory primordium.

### Dorso-ventral gene expression patterning during tracheo-oesophageal separation

Previously, we described a ventral-to-dorsal shift in expression of *Shh*, just rostral to the wave of separation as it passes up the normal foregut (Ioannides et al., 2003). At E10.5, the ventral ‘tracheal’ foregut domain is positive for *Shh*, while the dorsal ‘oesophageal’ domain is negative. However, by E11.5, the expression pattern is reversed so that the dorsal domain is now *Shh*-positive, while the ventral domain is *Shh*-negative. Importantly, Adriamycin-treated embryos with faulty tracheo-oesophageal separation fail to exhibit this ventral-dorsal shift: the undivided foregut exhibits relatively uniform *Shh* expression along the dorso-ventral axis, at both E10.5 and E11.5, and lacks a clear dorso-ventral expression boundary (Ioannides et al., 2003). To determine whether disturbance of dorso-ventral patterning is an obligatory feature of OA/TOF, we performed immunohistochemistry for the ventrally restricted foregut marker Nkx2.1 (Minoo et al., 1999) and *in situ* hybridisation for the dorsal marker *Sox2* (Que et al., 2007) in both normal and Adriamycin-treated embryos.

#### Normal development

**Fig. 3:**
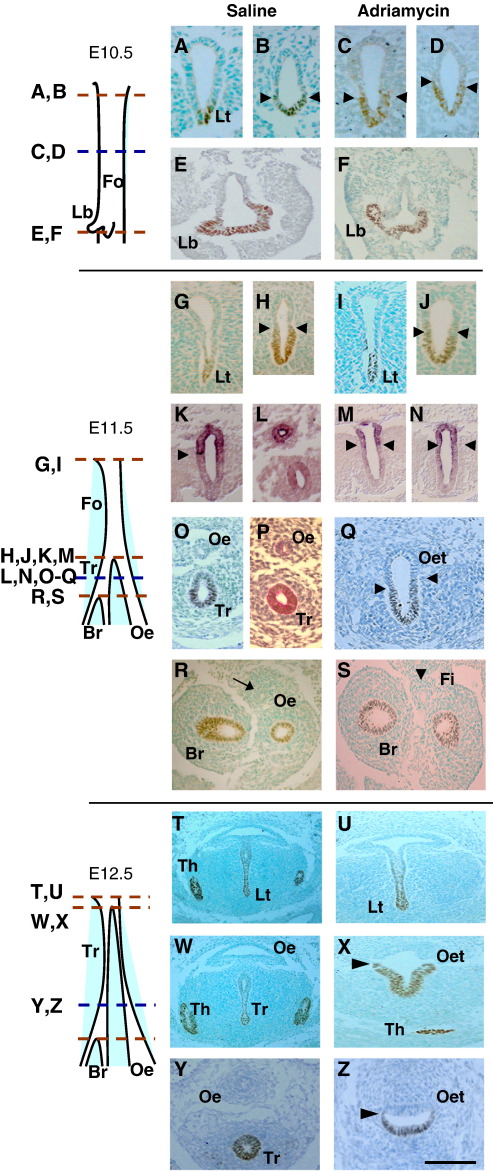
Dorso-ventral patterning in the foregut during tracheo-oesophageal separation. (A–Z) Immunohistochemistry for the respiratory marker Nkx2.1, except for (K–N) which show *in situ* hybridisation for *Sox2*, and (T) which is stained with respiratory-specific Periodic Acid Schiff. Transverse sections are from embryos at E10.5 (A–F), E11.5 (G–S) and E12.5 (T–Z), at levels indicated by the schematic representations. Red dotted lines on schematics define the levels for measuring foregut lengths as in Fig. 2. Arrow in (R) points to oesophagus, arrowhead in (S) points to fistula, and all other arrowheads indicate dorso-ventral expression boundaries. Abbreviations: Br, bronchus; Fi, fistula; Fo, foregut; Lb, lung bud; Lt, laryngotracheal groove; Oe, oesophagus; Oet, oesophagotrachea; Th, thyroid; Tr, trachea. Scale bar (in Z): 125 μm in A–Q; 80 μm in R,S; 100 μm in T–Z.

We confirmed that Nkx2.1 protein is restricted to the ventral, future respiratory foregut of both E10.5 and E11.5 embryos, at all rostro-caudal levels including the laryngotracheal groove (Figs. 3A, G), the undivided foregut (Figs. 3B, H) and the lung buds (Figs. 3E, R). Periodic Acid Schiff (PAS), a marker of mucopolysaccharide-rich respiratory epithelium (ten Have-Opbroek et al., 1991), demonstrates that Nkx2.1 expression coincides with the pattern of respiratory cell differentiation in the foregut endoderm (Figs. 3O, P). At E12.5, when tracheo-oesophageal separation is almost complete, the ventral foregut including infraglottic larynx (Fig. 3T) and trachea (Figs. 3W, Y) remain strongly positive for Nkx2.1, whereas the oesophagus is negative (Figs. 3W, Y). In contrast, *Sox2* transcripts are dorsally located in the normal foregut, both prior to and following tracheo-oesophageal separation, with a sharp expression boundary between dorsally positive and ventrally negative domains (Figs. 3K, L). This boundary appears to be closely similar to that respected by Nkx2.1 (compare Figs. 3H and K).

#### Adriamycin-induced OA/TOF

Adriamycin-treated embryos are comparable in stage to untreated control embryos as judged by somite number at E10.5 and E11.5 (Supplementary Fig. 1A). Moreover, early developmental events (E10.5) within the anterior foregut are not overtly disturbed by Adriamycin treatment, as judged by morphology and Nkx2.1 expression at rostral (compare Figs. 3A, C), intermediate (Figs. 3B, D) and caudal (Figs. 3E, F) levels. Tracheo-oesophageal development first diverges from normal at E11.5 when around 40% of Adriamycin-treated embryos fail to initiate foregut separation (compare Figs. 3O, Q). In these affected embryos, the undivided oesophago-trachea gives rise to an Nkx2.1-negative foregut connection (fistula) to the stomach (Fig. 3S). Despite this abnormality of foregut morphogenesis, Nkx2.1 continues to mark the respiratory elements of the Adriamycin-treated foregut in an appropriate dorso-ventral manner. Hence, the endoderm of the laryngotracheal groove expresses Nkx2.1 at E11.5 (Fig. 3I) and E12.5 (Fig. 3U), and the ventral, respiratory part of the undivided foregut remains Nkx2.1 positive, separated by a sharp boundary from the dorsal Nkx2.1-negative oesophageal domain at E11.5 (Fig. 3Q) and E12.5 (Figs. 3X, Z). The lung buds remain strongly Nkx2.1-positive (Fig. 3S).

The dorsal restriction of *Sox2* expression is also maintained in the foregut of affected E11.5 Adriamycin-treated embryos, both at the level where normal embryos have an undivided foregut (Fig. 3M) and more caudally where normal tracheo-oesophageal separation is complete but Adriamycin-treated embryos exhibit an undivided oesophago-trachea (Fig. 3N). At both rostro-caudal levels, the boundary between dorsally positive and ventrally negative *Sox2* domains appears closely similar to that demarcating dorsally negative and ventrally positive Nkx2.1 domains (compare Fig. 3J with M, N).

In conclusion, both Nkx2.1 and *Sox2* exhibit apparently normal dorso-ventral patterning of gene expression in the undivided Adriamycin-treated foregut, in contrast to the dynamic ventral-dorsal shift of *Shh* expression which is abolished in embryos with faulty tracheo-oesophageal separation.

### Failure of tracheo-oesophageal separation in *Shh* null embryos

Foregut malformations have been described in embryos with loss of *Shh* function (Litingtung et al., 1998), although the developmental basis of the defects has not been determined. We confirmed that *Shh* null embryos are growth retarded, and have multiple malformations affecting forebrain, heart and limbs (Supplementary Figs. 2A, B). However, somite number, a measure of developmental stage at E10.5 and E11.5, does not differ significantly with genotype in *Shh* litters (Supplementary Fig. 2C), validating a comparison of foregut morphology between *Shh* null embryos and their normally developing litter mates.

**Fig. 4:**
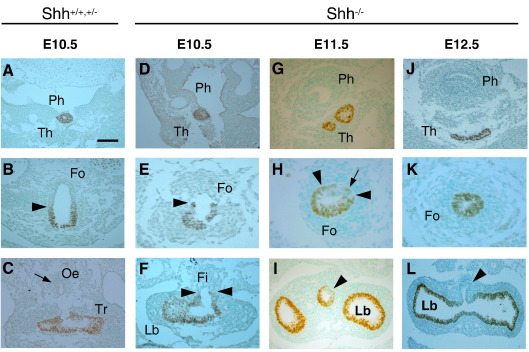
Respiratory specification in *Shh* null embryos. *Nkx2.1* expression detected by immunohistochemistry in pooled wild type and heterozygous (A–C) and *Shh* null (D–L) embryos. (A, D, G, J) Pharyngeal level, at which there is comparable expression in the thyroid primordium. (B, E, H, K) Intermediate foregut level at which the dorso-ventral Nkx2.1 boundary is undisturbed in E10.5 *Shh* null embryos (arrowheads in B, E). An Nkx2.1-negative domain is still present in the *Shh* null at E11.5 (arrowheads and arrow in H) but is not evident at E12.5 (K). (C, F, I, L) Caudal end of the respiratory foregut, where *Shh* null embryos have a well-defined fistula that is either wholly (arrowheads in F, L) or partly (arrowhead in I) Nkx2.1-negative. Note also the grossly abnormal, unbranched and fused lung buds (F,L). This contrasts with the clearly separate trachea and oesophagus (arrow in C) of normally developing embryos. Abbreviations as in Fig. 3 and: Ph, pharynx. Scale bar (in A): 100 μm in A, B, C, D, G; 50 μm in E, J; 67 μm in F, I, L; 33 μm in H, K.

At E10.5, tracheo-oesophageal separation has just started at the level of the lung buds in wild type littermates (Fig. 4C), thus occurring 12 h earlier than in untreated CBA/Ca embryos, presumably as a result of the difference in genetic background. In contrast, *Shh*^−/−^ embryos at this (Fig. 4F) and subsequent stages do not exhibit foregut division. Nevertheless, Nkx2.1 expression in E10.5 *Shh*^−/−^ embryos closely resembles that of wild type littermates, with an Nkx2.1-positive thyroid primordium (Figs. 4A, D) and a well-defined boundary between Nkx2.1 expressing and non-expressing cells (Figs. 4B, E). At the caudal end of the undivided foregut there is a trifurcation of two Nkx2.1-positive lung buds and an Nkx2.1-negative fistula (Fig. 4F). Hence, the appearance in E10.5 *Shh*^−/−^ embryos is closely similar to that seen in affected E11.5 Adriamycin-treated embryos.

At E11.5 and E12.5, the appearance of the undivided *Shh*^−/−^ foregut diverges from that seen in the Adriamycin model. The *Shh*^−*/*−^ foregut narrows markedly and well-defined tracheal and oesophageal domains, as judged by Nkx2.1 expression, are no longer evident (Figs. 4H, K). Moreover, the intensity of expression of Nkx2.1 in the undivided foregut appears weaker than in either the thyroid primordium or lung buds (compare Figs. 4H, K with Figs. 4G, I, J, L). Although most of the *Shh*^−*/*−^ undivided foregut endoderm is Nkx2.1-positive, a narrow non-expressing domain persists dorsally (arrowheads and arrow in Fig. 4H) giving rise, at more caudal levels, to a partially or wholly Nkx2.1-negative fistula (arrowheads in Figs. 4I, L).

Hence, at the stage when foregut separation is beginning in normal embryos, both morphology and Nkx2.1 expression pattern are closely similar in the undivided foregut of Adriamycin-treated and *Shh* mutant embryos. Considering that *Shh* null embryos fail to divide the foregut, and that failed separation in the Adriamycin-treated embryos is associated with disruption of *Shh* expression pattern, it also seems likely that the dynamic ventral-dorsal shift in *Shh* signalling is required for the onset of tracheo-oesophageal separation.

### Cell proliferation during normal tracheo-oesophageal separation

To investigate the cellular mechanisms of tracheo-oesophageal separation, we examined cell proliferation in the early foregut. No significant difference in the percentage of cells staining positive (H3-index) for the mitotic marker phosphohistone H3 was detected along the dorso-ventral axis of the untreated CBA/Ca foregut. This argues against a role for localised cell proliferation in normal tracheo-oesophageal separation, and reinforces the conclusion that outgrowth specifically of the ventral foregut component is unlikely to underlie tracheal development in mice. At E11.5, the undivided foregut had significantly lower H3-indices than the divided foregut (Supplementary Fig. 3), in agreement with our finding (Fig. 2A) that growth of the entire foregut is most rapid after separation is complete.

### Programmed cell death (PCD) during normal tracheo-oesophageal separation

**Fig. 5:**
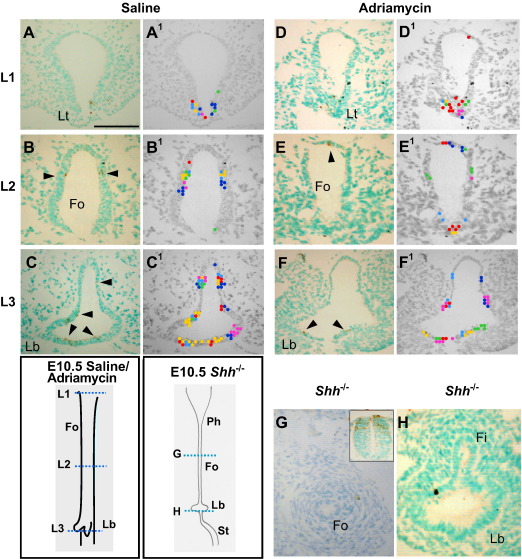
PCD in the E10.5 foregut: effect of Adriamycin and loss of *Shh* function. (A–H) Immunohistochemistry for activated caspase 3 on saline-treated (A–C; *n* = 6), Adriamycin-treated (D–F; *n* = 6) and *Shh* null (G,H; *n* = 3) embryos at E10.5. Transverse sections are from levels indicated on the schematic representations. Arrowheads indicate caspase 3-positive (brown stained) apoptotic cells. (A^1^–F^1^) Templates used to illustrate spatial PCD patterns. Dots of different colour represent individual apoptotic cells from different embryos and are marked at their equivalent positions on the template. Note the lack of caspase 3-positive cells in the lateral walls of the Adriamycin-treated foregut (E, E^1^) compared with the saline control (B, B^1^). There is a paucity of PCD in the *Shh* null foregut (H), despite the clear occurrence of dying cells in the dorsal neural tube of this embryo (inset in G). Abbreviations as in Fig. 2, and: St, stomach. Scale bar (in A): 100 μm for all parts.

**Fig. 6:**
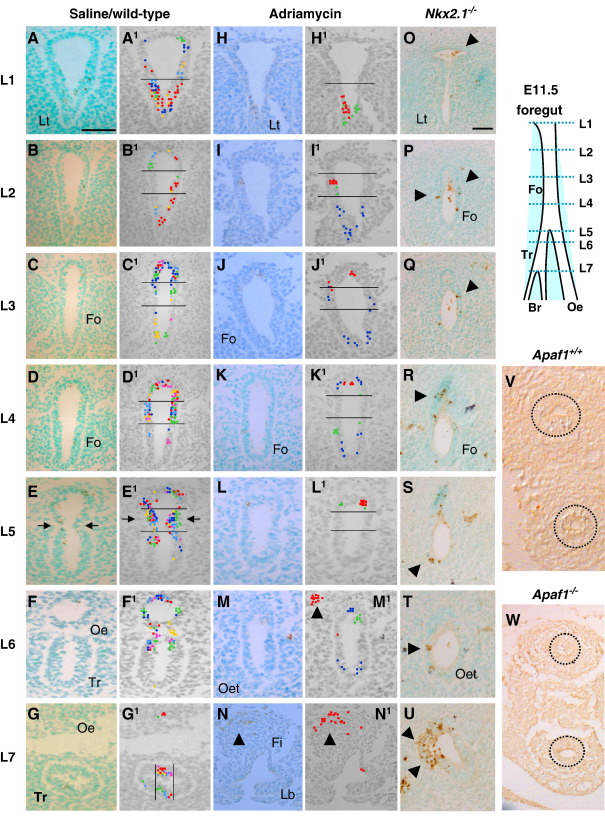
PCD during tracheo-oesophageal separation at E11.5 and E12.5: effect of Adriamycin and loss of *Nkx2*.1 or *Apaf1* function. (A–W) Immunohistochemistry for activated caspase 3 on transverse sections through the foregut of E11.5 saline-treated (A–G; *n* = 6), Adriamycin-treated (H–N; *n* = 6) and *Nkx2.1*^−/−^ (O–U; *n* = 3) embryos, and of E12.5 *Apaf1*^*+/+*^ and *Apaf1*^−*/*−^ embryos (V,W; *n* = 3). Sections are from levels indicated on the schematic representation. (A^1^–N^1^) Templates used to illustrate spatial PCD patterns as described in Fig. 5. Horizontal lines define foregut zones for calculation of PCD index (see Fig. 7). Arrows in (E, E^1^) indicate the concentration of apoptotic cells in epithelial ridges immediately cranial to the point of foregut separation, a finding that is not seen in either Adriamycin-treated (L, L^1^) or *Nkx2.1*^−/−^ (R–T) embryos. Arrowheads in (M^1^,N) show apoptotic cells in the dorsal mesenchyme associated with a cellular condensation. Arrowheads in (O–U) highlight excessive apoptosis in *Nkx2.1*^−/−^ embryo. Circles in (V,W) highlight the separated trachea and oesophagus in *Apaf1*^*+/+*^ and *Apaf1*^−*/*−^ embryos. Abbreviations as in Figs. 3, 4 and5. Scale bar (in A): 50 μm for all parts.

To determine whether PCD might play a role in foregut separation, we used immunohistochemistry for activated caspase-3 to identify apoptotic cells in transverse sections (Figs. 5 and 6). A templating technique (Sharma et al., 2004) enabled individual apoptotic cells from equivalent levels of replicate embryos to be marked on a single template section. This helped in discerning spatial PCD patterns, despite the small overall number of caspase-3 positive cells. In contrast to mitotic activity, considerable variation in PCD activity was detected along both the rostro-caudal and dorso-ventral axes of the foregut.

At E10.5, three sites of particularly intense epithelial PCD were distinguished in normal embryos: in the laryngotracheal groove at the rostral end of the foregut (Figs. 5A, A^1^), at the dorso-ventral boundary of the undivided foregut walls, more caudally (Figs. 5B, B^1^), and at the sites of budding of the broncho-pulmonary primordia, most caudally (Figs. 5C, C^1^). At E11.5, a similar concentration of PCD was observed in the laryngotracheal groove (Figs. 6A, A^1^) and broncho-pulmonary buds (Fig. 6G,G^1^). Notably, at intermediate rostro-caudal levels there was a marked ventral-to-dorsal shift of PCD, both in the undivided (Figs. 6B–E, B^1^–E^1^) and divided (Figs. 6F, F^1^) foregut. At the point of tracheo-oesophageal separation, we detected a marked increase in the number of dying cells at the point of separation (Figs. 6E, E^1^ arrows), a pattern that was maintained for a few sections caudal to the level of separation (Figs. 6F, F^1^).

**Fig. 7:**
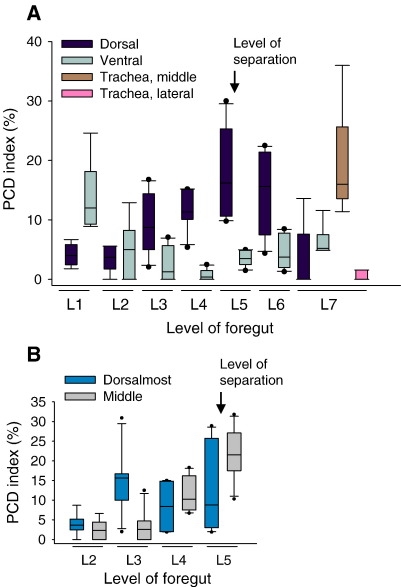
Quantification of PCD in the E11.5 foregut. Box plots showing PCD indices from E11.5 saline-treated CBA/Ca mice at different rostro-caudal levels. Dorso-ventral zones are defined as in Fig. 6. Arrows indicate level at which foregut separation has occurred at this stage. (A) PCD data for dorso-ventral zones with additional data (brown and pink boxes) for the middle and lateral tracheal segments to illustrate budding of the bronchi. (B) PCD data for dorsal-most and middle segments at levels 2 to 5. Note ventral-to-dorsal switch in predominance of PCD as separation proceeds from caudal to rostral within the foregut (compare black and grey bars in A). The point of foregut separation coincides with a peak of PCD (A), with middle dorso-ventral zone showing greatest intensity of apoptosis (B).

We quantified PCD by determining the proportion of caspase-3-positive cells (PCD index) in dorso-ventral zones of the foregut. A striking concentration of PCD was observed in the ventral laryngotracheal groove at rostro-caudal level 1 (L1), with up to 25% of cells dying (Fig. 7A). In contrast, ventral regions of the foregut more caudally (L3,4,5) showed significantly less PCD (*p* < 0.004). In the dorsal half of the foregut, L1 and L2 had a low PCD index, whereas levels caudal to L2 exhibited markedly more apoptosis, with a peak at L5–6, where as many as 25–30% of dorsal epithelial cells are dying (Fig. 7A). Since L5 is the level at which tracheo-oesophageal separation is actively occurring, we sub-divided the dorsal zone into dorsal-most and middle zones, to determine whether PCD is particularly associated with foregut wall invagination (middle zone). While PCD is low in the middle zone rostrally, at L2 and L3 (Figs. 6B^1^, C^1^), PCD index rises sharply at L4 (Fig. 6D^1^) and peaks at L5 (Fig. 6E^1^), where up to 32% of cells are dying (Fig. 7B). The dorsal-most segment also has high levels of PCD, but this is also present at L3, more rostrally. Hence, the two main characteristics of PCD in the normal E11.5 foregut are a ventral-dorsal shift, and a concentration of dying cells at the point of tracheo-oesophageal separation.

### PCD in models of abnormal tracheo-oesophageal development

The PCD pattern in Adriamycin-treated embryos is similar at both E10.5 and E11.5. Dying cells localise to the laryngotracheal groove (Figs. 5D, D^1^ and 6H, H^1^), and to the origin of the broncho-pulmonary buds (Figs. 5F, F^1^), as in saline control embryos. In contrast, the dorso-ventral pattern of PCD localisation in the Adriamycin-treated foregut is disrupted at intermediate rostro-caudal levels, both prior to the onset of tracheo-oesophageal separation at E10.5, and while separation is ongoing at E11.5. Only one out of six Adriamycin-treated embryos at E10.5 exhibited PCD clusters around the dorso-ventral boundary, as seen in control embryos, with the remaining embryos showing apoptotic cells at apparently random points along the foregut dorso-ventral axis (Figs. 5E, E^1^).

Similar variability in dorso-ventral PCD location was seen in E11.5 Adriamycin-treated embryos with failed foregut separation (Figs. 6I–M and I^1^–M^1^). Indeed, there is a striking lack of PCD in the middle foregut zone (Figs. 6K, K^1^, L, L^1^), whereas PCD clusters are more frequently seen in the dorsal-most foregut zone. Apoptotic cells also occur in the mesenchyme dorsal to the undivided foregut, in association with an apparent mesenchymal condensation at this site (Figs. 6M^1^, N, N^1^ arrowheads), whereas significant PCD was not observed elsewhere in the mesenchyme around the foregut.

*Shh* null embryos at E10.5, when wild type and heterozygous littermates are already undergoing tracheo-oesophageal separation, resemble Adriamycin-treated embryos in lacking PCD localisation at the dorso-ventral boundary of the foregut. Occasional apoptotic cells occur apparently randomly along the dorso-ventral foregut axis. Furthermore, the overall intensity of foregut PCD appears markedly reduced in *Shh* mutants, compared to normally developing littermates (Figs. 5G, H), a striking finding in view of the excessive apoptosis seen, for example, in the neural tube of *Shh*^−*/*−^ embryos (Fig. 5G inset).

We also examined PCD in another model of OA/TOF: embryos null for *Nkx2.1* exhibit failure of tracheo-oesophageal separation (Minoo et al., 1999; Yuan et al., 2000). Wild type litter mates at E11.5 exhibit a pattern and intensity of apoptosis closely resembling CBA/Ca embryos (data not shown). In contrast, we found excessive PCD in the foregut of *Nkx2.1*^−*/*−^ embryos (Figs. 6O–U). Large numbers of caspase 3-positive cells were observed at all rostro-caudal levels, with no apparent dorso-ventral localisation. So plentiful was this PCD that it could be readily visualised without use of the templating technique.

In conclusion, we have identified a disturbed pattern of foregut PCD in three different models of OA/TOF. In particular, the concentration of dying cells at the point of tracheo-oesophageal separation is lost in all three cases. There is great variation, however, in the intensity of apoptosis: Adriamycin-treated embryos have relatively normal numbers of dying cells, *Shh*^−*/*−^ embryos show reduced foregut apoptosis, and *Nkx2.1*^−*/*−^ embryos exhibit excessive apoptosis. These findings could indicate a potentially important role for the spatial regulation of PCD during the process of tracheo-oesophageal separation. However, we cannot rule out the alternative view, that PCD could be a secondary consequence of the ongoing morphogenesis.

### Normal foregut morphogenesis in *Apaf1* mutant embryos lacking PCD

To determine the requirement for PCD in tracheo-oesophageal separation, we took a genetic approach by examining embryos null for *Apaf1*, the homologue of CED-4/ARK which is required for activation of caspase-9. The lack of Apaf-1 results in reduced activation of caspase-3 and dramatically reduced PCD (Cecconi et al., 1998). Hence, if PCD plays a functional role in tracheo-oesophageal separation, we would expect to find abnormalities in the septation process in *Apaf-1*^−*/*−^ embryos. At E11.5, the morphology of foregut separation did not appear different between *Apaf1*^−*/*−^ embryos and their wild type litter mates (data not shown). Moreover, at E12.5, we observed a completely separated oesophagus and trachea in all *Apaf1* mutant and wild type embryos examined, despite an apparent complete suppression of apoptosis (Figs. 6V, W). Hence, it appears that the genetically determined lack of apoptosis, which leads to defects in several other developmental systems in *Apaf1*^−*/*−^ embryos (Cecconi et al., 1998, 2004), does not disrupt tracheo-oesophageal separation. We conclude that PCD is not essential for foregut separation, despite its close spatio-temporal correlation with this morphogenetic process.

## Discussion

In the present study, we compared normal development of the trachea and oesophagus with abnormal development in three mouse models (Adriamycin, *Shh* and *Nkx2.1*) that exhibit malformations resembling human OA/TOF. Despite the difference in causation of these models, the morphological and molecular features of abnormal early foregut development are remarkably similar. This raises the possibility that human OA/TOF malformations may also originate from a developmental pathogenesis like that seen in the mouse models.

### Does the respiratory foregut arise by septation or tracheal outgrowth?

Previous studies in both normal embryos and in the Adriamycin rat model have generated contrasting theories to explain the origin of a separate tracheal structure from the common foregut, and failure of this process in OA/TOF. The ‘tracheal outgrowth’ model views growth of the respiratory primordium as primarily responsible for emergence of the trachea as a separate structure. According to this idea, failure of tracheal growth is likely to be the primary cause of OA/TOF (Sasaki et al., 2001; Zaw-Tun, 1982). In contrast, the ‘septation’ model views the formation of a septum across the foregut tube as the critical event in tracheo-oesophageal separation, with defective caudal–rostral propagation of septum formation being the primary defect leading to OA/TOF. In the present study, we measured the length of the respiratory foregut, and of its divided and undivided components, prior to, during and following formation of the trachea as a distinct embryonic structure. The analysis clearly demonstrates that while total respiratory foregut length increases linearly, this is independent of tracheo-oesophageal separation, with similar dynamics in affected Adriamycin-treated embryos as in untreated controls. Moreover, as the length of divided foregut increases in normal embryos, so the absolute length of undivided foregut decreases, a finding that is not predicted by the tracheal outgrowth model. Finally, in an analysis of cellular proliferation, we did not detect an enhanced rate of growth in the ventral foregut at the time of origin of the trachea, as demanded by the tracheal outgrowth hypothesis. Therefore, while rapid overall growth of the foregut is an integral part of tracheo-oesophageal morphogenesis, our findings are more consistent with the ‘septation’ theory of tracheal development than with a model that invokes specific outgrowth of the trachea.

In support of this conclusion are reports that bilateral epithelial ridges, or invaginations, can be observed approaching each other in the midline just rostral to (i.e. preceding) the point of tracheo-oesophageal separation (Qi and Beasley, 2000; Williams et al., 2003). We also observed bilateral epithelial ridges undergoing fusion leading to separation of the tracheal and oesophageal tubes. These structures were absent or morphologically abnormal in affected Adriamycin-treated embryos, and in *Shh*^−*/*−^ and *Nkx2.1*^−*/*−^ mutant embryos. Hence, lack of formation of lateral epithelial ridges correlates with failed separation of the trachea and oesophagus in several systems, making it seem likely that fusion of the lateral epithelial ridges leads to septation of the normal foregut tube. Future studies using high-resolution, real time imaging of the separating foregut should provide further information on this point.

### Is defective foregut separation associated with disturbance of dorso-ventral gene expression patterning?

We next examined an unresolved issue relating to the role of disturbed dorso-ventral gene expression patterning in failed tracheo-oesophageal separation. Faulty separation occurs in mice lacking either the dorsal foregut marker, *Sox2*, or the ventral foregut marker, *Nkx2.1* (Minoo et al., 1999; Que et al., 2007). Moreover, *Sox2* is ectopically expressed in the ventral foregut of *Nkx2.1* mutants and, conversely, *Nkx2.1* is ectopically expressed in the dorsal foregut of *Sox2* mutants. These findings have led to the idea that disturbed foregut dorso-ventral patterning may be a primary mechanism leading to failure of tracheo-oesophageal separation (Minoo et al., 1999; Que et al., 2007). We examined this question in the Adriamycin mouse model of OA/TOF and found that failure of foregut septation is not associated with a generalised loss of dorso-ventral gene expression patterning in the foregut. Both Nkx2.1 and *Sox2* show appropriate patterning as Adriamycin-induced OA/TOF develops. Nkx2.1 is also correctly patterned in the undivided *Shh*-null foregut. On the other hand, the dynamic ventral-to-dorsal shift in expression of *Shh*, which occurs just ahead of the caudal–rostral wave of foregut separation, is abolished in Adriamycin-induced foregut defects (Ioannides et al., 2003). This is consistent with previous findings of a reduced level of Shh mRNA and protein in the rat Adriamycin-treated foregut (Arsic et al., 2003; Arsic et al., 2004), and with failure of downregulation of *Shh* at the site of tracheo-oesophageal separation (Orford et al., 2001). Moreover, reduced levels of *Gli2*, a key member of the Shh signalling cascade, have been found in the mesenchyme of the fistula in Adriamycin-treated rats (Spilde et al., 2003b), and in human neonates with OA/TOF (Spilde et al., 2003a). Hence, from our analysis it appears that the spatio-temporal shift in *Shh* expression is critical for normal tracheo-oesophageal separation.

We found that the fistula arising from the dorsal part of the undivided, Adriamycin-treated foregut is Nkx2.1-negative at E11.5. In contrast, the fistula of the Adriamycin rat is reported to be Nkx2.1-positive at E13.5 (Crisera et al., 1999) while the fistula in the *Shh* null mouse was found to have tracheal characteristics at E17.5 (Litingtung et al., 1998). It seems likely that progressive ‘trachealisation’ of the undivided foregut, as described in the Adriamycin rat model (Kluth et al., 1987; Merei et al., 1998; Possögel et al., 1998), may account for this difference in findings. Hence, there appears to be a gradual extension of Nkx2.1 expression into the fistula as a secondary, adaptive event, converting the initially Nkx2.1-negative trifurcation branch at E11.5 into an Nkx2.1-positive fistula from E13.5 onwards. Nevertheless, the absence of tracheal cartilage from the oesophagotrachea of Adriamycin-treated fetuses suggests that this process of trachealisation may not progress to completion.

### Localised cell proliferation and apoptosis—a role in tracheo-oesophageal septation?

A third aim of the present study was to ask whether localised cell proliferation may be involved in formation of the bilateral epithelial ridges, or whether apoptosis may play a functional role in the fusion and remodelling of the epithelial ridges as the trachea and oesophagus separate. While we detected active cell proliferation in the epithelium and underlying mesenchyme, there was no evidence of proliferation ‘hot spots’ in the dorso-ventral foregut axis, that might be causally related to epithelial ridge formation. Hence, localised cell proliferation does not appear to be a significant factor in foregut separation.

In our analysis of apoptosis, dying cells were found to comprise up to 30% of the lateral epithelial ridges, just prior to formation of the septum and for a short period following tracheo-oesophageal separation. This association of apoptosis with the lateral epithelial ridges was disrupted in each of the three models of defective foregut separation. Whereas Adriamycin-treated embryos showed a relatively normal overall intensity of PCD, *Shh*^−*/*−^ and *Nkx2.1*^−*/*−^ embryos exhibited diminished and excessive apoptosis respectively. Previous studies have reported reduced apoptosis in the region of defective tracheo-oesophageal separation in the Adriamycin rat model (Orford et al., 2001; Qi and Beasley, 2000; Williams et al., 2000; Zhou et al., 1999). Hence, considerable evidence exists to link PCD spatio-temporally with morphogenetic foregut division.

To investigate whether apoptosis is functionally involved in foregut separation, we studied the *Apaf1* null mouse in which PCD is reduced to extremely low levels (Cecconi et al., 1998). Strikingly, despite an apparent complete suppression of apoptosis in *Apaf*^−*/*−^ embryos, we found that tracheo-oesophageal separation proceeds to completion, with the formation of a normally separated oesophagus and trachea. We conclude that apoptosis, although plentiful in the vicinity of the foregut epithelial ridges, is not functionally required for separation. This finding provides a strong parallel with our recent analysis of apoptosis in mouse neural tube closure (Massa et al., 2009) and with work on palatal shelf closure (Takahara et al., 2004). In both of these studies, apoptosis was shown to be spatio-temporally correlated with the closure event, whereas experimental analysis failed to identify an essential role for PCD in completion of morphogenesis. In contrast, other studies have identified an apparent functional role for apoptosis in chick neurulation (Weil et al., 1997) and mouse palatal closure (Cuervo et al., 2002), indicating that this remains a research area of some controversy.

### Foregut epithelial remodelling and the origin of apoptotic cells

Disruption of the epithelial layer covering the fusing bilateral foregut ridges, and epithelial reformation to create separate tracheal and oesophageal tubes, are essential events for tracheo-oesophageal septation. Similar epithelial remodelling processes occur in other morphogenetic fusion events, including neurulation and palatal closure, each with its closely associated apoptosis. If cell death is not required for epithelial remodelling, as is suggested by our data, then it seems most likely that apoptosis is a secondary consequence of the morphogenesis. Indeed, it is well know that cells which lose adhesive contacts with their neighbours, or with the underlying extracellular matrix, are prone to undergoing apoptosis, a process termed ‘anoikis’ (Gilmore, 2005). In the neural folds, the Nf2 tumour suppressor (also called Merlin) regulates the assembly and maintenance of apico-lateral junctional complexes and is required for the avoidance of anoikis (McLaughlin et al., 2007). It seems possible, therefore, that fusion of the lateral epithelial ridges and the associated epithelial remodelling results in a proportion of cells transiently losing their cell–cell or cell–matrix contacts. These cells undergo anoikis, producing the apoptosis that is associated with normal tracheo-oesophageal separation.

### Alternative mechanisms of epithelial ridge formation and fusion

Since neither localised cell proliferation nor apoptosis appear implicated in the mechanism of foregut separation, it is important to consider alternative cellular processes that can lead to deformation of epithelial sheets during morphogenesis. For example, contraction of apically located actin microfilaments is essential for the elevation and bending of the rostral neural folds (Morriss-Kay and Tuckett, 1985; Sadler et al., 1986; Ybot-Gonzalez and Copp, 1999), and constrains the direction of expansion of the pharyngeal pouches (Quinlan et al., 2004). However, the epithelial foregut ridges comprise convex bulges into the lumen and, to achieve this epithelial deformation, contracting microfilaments would need to be localised basally, rather than apically as in other systems. Another mechanism that might be implicated in epithelial ridge formation is localised expansion of the mesenchyme underlying the lateral foregut epithelium, as observed in the mouse cranial neural folds which accumulate a hyaluronan-rich extracellular matrix (Morris-Wiman and Brinkley, 1990; Solursh and Morriss, 1977). However, in the present study, we did not observe obvious alterations in intercellular space within the mesenchyme of the lateral epithelial ridges. Clearly, there is considerable scope for further studies of the cellular basis of foregut separation in mammalian embryos.
