# Supplementary material for: Foregut separation and tracheo-oesophageal malformations: The role of tracheal outgrowth, dorso-ventral patterning and programmed cell death
Source: Dev Biol. 2010 Jan 15;337(2):351–62. doi: 10.1016/j.ydbio.2009.11.005 (PMC2877773; doi:10.1016/j.ydbio.2009.11.005)
Supplement: Supplementary data [file mmc1.ppt]

## Slide 1
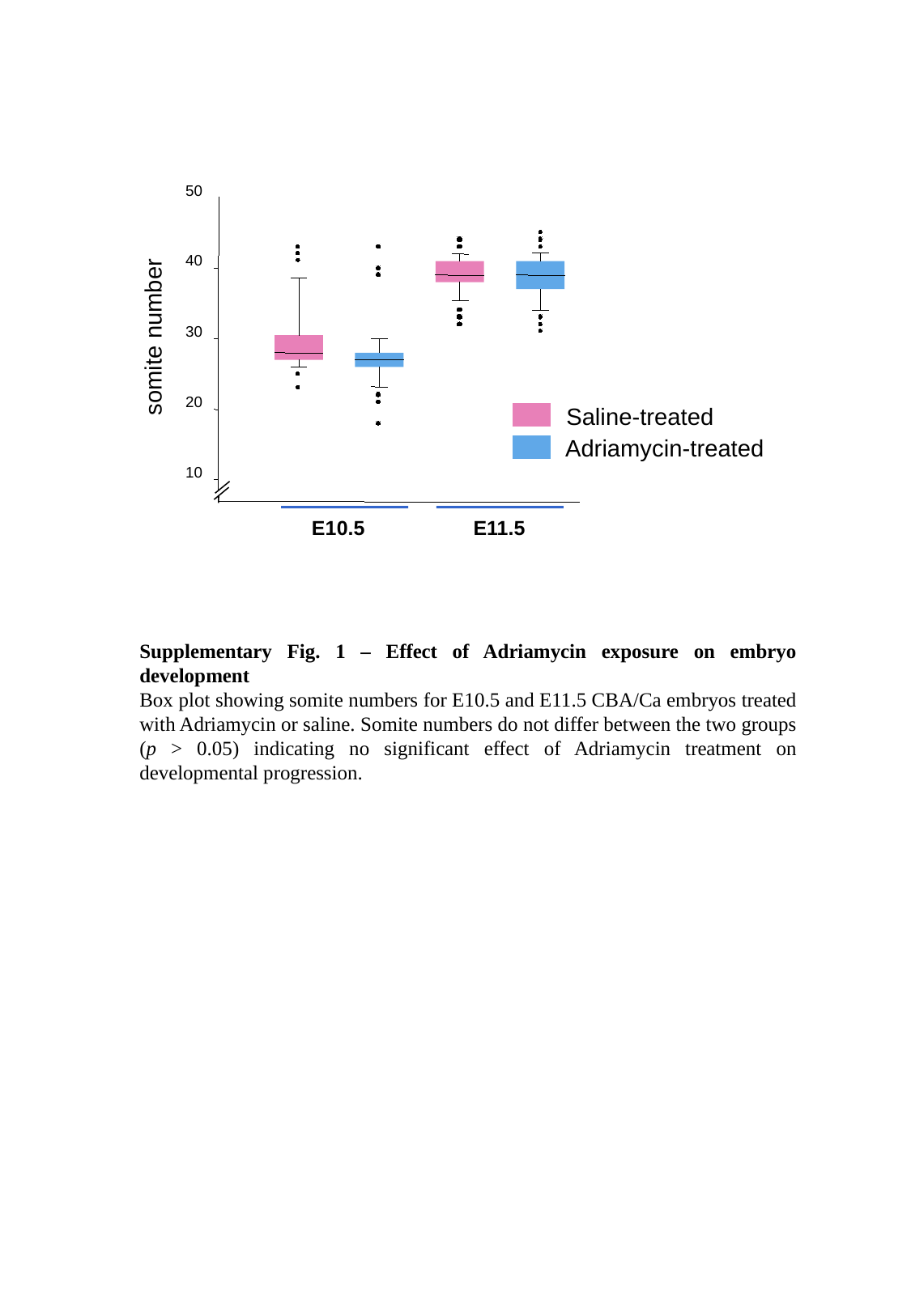

50
40
somite number
30
20
Saline-treated
Adriamycin-treated
10
E10.5
E11.5
Supplementary Fig. 1 – Effect of Adriamycin exposure on embryo development
Box plot showing somite numbers for E10.5 and E11.5 CBA/Ca embryos treated with Adriamycin or saline. Somite numbers do not differ between the two groups (p > 0.05) indicating no significant effect of Adriamycin treatment on developmental progression.

## Slide 2
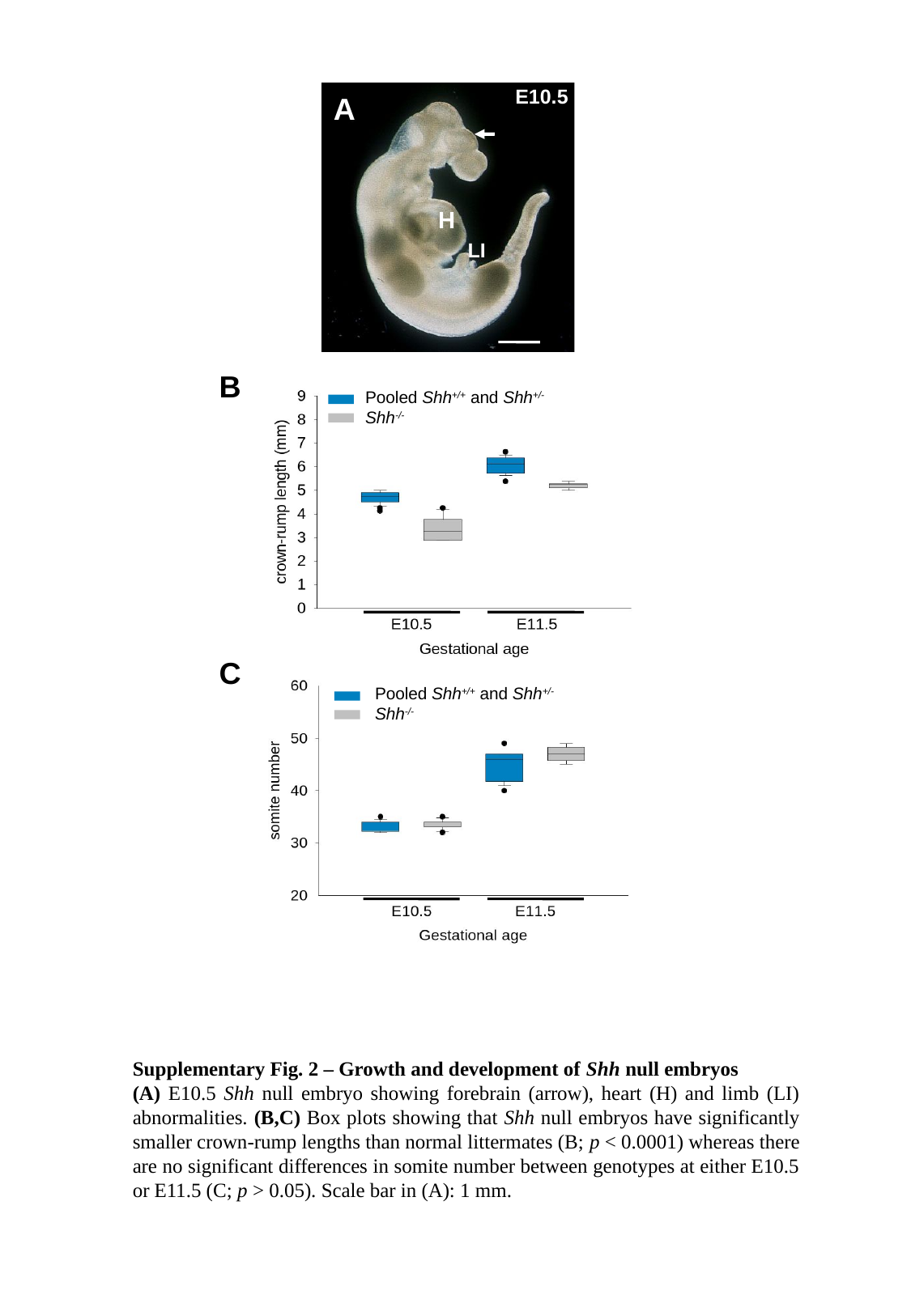

E10.5
A
H
LI
B
Pooled Shh+/+ and Shh+/-
Shh-/-
C
Pooled Shh+/+ and Shh+/-
Shh-/-
Supplementary Fig. 2 – Growth and development of Shh null embryos
(A) E10.5 Shh null embryo showing forebrain (arrow), heart (H) and limb (LI) abnormalities. (B,C) Box plots showing that Shh null embryos have significantly smaller crown-rump lengths than normal littermates (B; p < 0.0001) whereas there are no significant differences in somite number between genotypes at either E10.5 or E11.5 (C; p > 0.05). Scale bar in (A): 1 mm.

## Slide 3
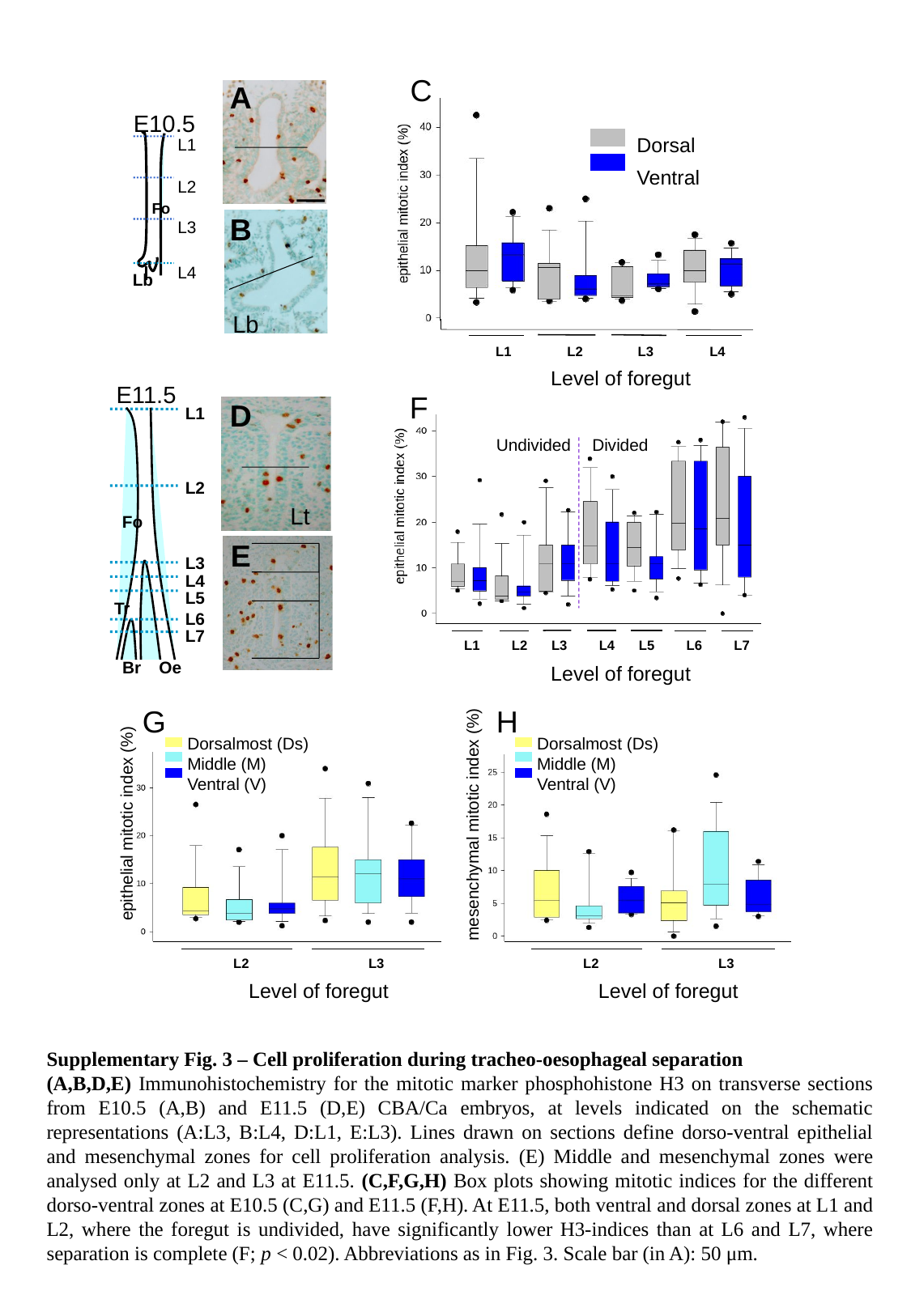

C
A
E10.5
L1
L2
Fo
L3
L4
Lb
Dorsal
Ventral
B
Lb
L1
L2
L3
L4
Level of foregut
E11.5
L1
L2
Fo
L3
L4
L5
Tr
L6
L7
Br
Oe
F
D
Lt
Undivided
Divided
E
L1
L2
L3
L4
L5
L6
L7
Level of foregut
G
H
Dorsalmost (Ds)
Middle (M)
Ventral (V)
Dorsalmost (Ds)
Middle (M)
Ventral (V)
epithelial mitotic index (%)
mesenchymal mitotic index (%)
L2
L3
L2
L3
Level of foregut
Level of foregut
Supplementary Fig. 3 – Cell proliferation during tracheo-oesophageal separation
(A,B,D,E) Immunohistochemistry for the mitotic marker phosphohistone H3 on transverse sections from E10.5 (A,B) and E11.5 (D,E) CBA/Ca embryos, at levels indicated on the schematic representations (A:L3, B:L4, D:L1, E:L3). Lines drawn on sections define dorso-ventral epithelial and mesenchymal zones for cell proliferation analysis. (E) Middle and mesenchymal zones were analysed only at L2 and L3 at E11.5. (C,F,G,H) Box plots showing mitotic indices for the different dorso-ventral zones at E10.5 (C,G) and E11.5 (F,H). At E11.5, both ventral and dorsal zones at L1 and L2, where the foregut is undivided, have significantly lower H3-indices than at L6 and L7, where separation is complete (F; p < 0.02). Abbreviations as in Fig. 3. Scale bar (in A): 50 μm.
